# The Polypill: From Promise to Pragmatism

**DOI:** 10.1371/journal.pmed.1001862

**Published:** 2015-08-11

**Authors:** Mark D. Huffman

**Affiliations:** Department of Preventive Medicine, Northwestern University Feinberg School of Medicine, Chicago, Illinois, United States of America

## Abstract

Mark Huffman asks what happened to the polypill to reduce cardiovascular risk, explores the promise it still holds, and considers how best to turn promise into reality.

**Summary Points**

- Starting with the promise and a brief history of the polypill, this article considers its current status and highlights five pragmatic issues to address before the polypill can become a mainstream strategy.
- Initial phase I and II trials have not been powered to detect differences in clinical outcomes, but there is high-quality evidence that fixed-dose combination therapy improves adherence by 44% (95% CI: 26% to 65%), with even greater effects in individuals with low baseline adherence.
- A strategy of age-only screening and mass treatment with polypills should be abandoned; instead, focus should shift to the secondary prevention of cardiovascular diseases.
- Outcome trials would influence the adoption of fixed-dose combination therapy for cardiovascular disease secondary prevention, but no such trials were performed before widespread adoption of combination therapy for HIV, malaria, and other diseases.

## Introduction

“Whatever happened to the polypill?” my colleagues ask me when told about the status of fixed-dose combination therapy for cardiovascular disease prevention. While the concept has always seemed straightforward, the implementation of this apparently simple concept has proved more challenging than anticipated. More than a decade after the concept was introduced, the state of the science has moved considerably forward, but perhaps not as brightly as initially projected.

## The Promise and a Brief History of the Polypill

A polypill, or fixed-dose combination therapy, is a familiar strategy for multidrug delivery for anyone who has ever taken a multivitamin. Combinations of drugs are widely available to treat a variety of diseases, including HIV, tuberculosis, and raised blood pressure, among others [1]. In 2001, Professor Richard Peto and others first outlined the concept of using the fixed-dose combination of aspirin, a statin, an angiotensin-converting enzyme (ACE) inhibitor, and a beta blocker for cardiovascular disease secondary prevention in low- and middle-income countries [2]. However, it was not until 2003 that the modern concept of the *Polypill* was born. There was a brilliant *BMJ* cover, almost like a movie poster, as well as the catchy name and the tantalizing models by Professors Nicholas Wald and Malcolm Law that promised the prevention of more than 80% of all cardiovascular disease deaths [3]. No longer just for poor countries, the concept was hailed as a panacea. It was going to be cheaper, simpler, and plain better than what had been used before, just like any great innovation. All that needed to be done was to give the Polypill to everyone over 50 years old. However, critics quickly argued that polypills would medicalize whole populations, detract from individual-level health behaviors and population-level interventions, and even widen health disparities [4]. The next step was to move from models to trials to evaluate the effects of polypills.

Since 2009, there have been 13 trials (*n* = 8,898) of fixed-dose combination therapy including at least one statin and one blood pressure lowering drug for cardiovascular disease prevention reported [5]. Designed as pharmacokinetic and pharmacodynamic studies, none of these initial trials were powered to detect a difference in outcomes, and no differences in fatal or nonfatal events have been demonstrated. Six different formulations have been included in these trials, including combinations without aspirin. Like other fixed-dose combinations, these trials demonstrate a robust and consistent effect on improving adherence across diverse settings. Among the four trials (*n* = 3,338 participants) of multiple fixed-dose combinations that measured adherence [6–9], fixed-dose combination increased adherence by 44% (95% CI: 26% to 65%) compared with usual care in patients with prevalent cardiovascular disease or high risk for cardiovascular disease (Table 1), though substantial heterogeneity (I^2^ = 79%) was present [10]. Data from the Single Pill to Avert Cardiovascular Events (SPACE) individual participant data meta-analyses demonstrate that individuals with low baseline adherence had the greatest improvements in adherence, from 17% at baseline to 74% at trial end (relative risk [RR] = 4.46 [95% CI: 3.72 to 5.36]), compared with participants who were already adherent at baseline (86% to 90%; RR = 1.04 [1.01 to 1.07]) [11].

**Table 1:** Differences in adherence between the intervention and control groups in fixed-dose combination therapy trials that report adherence [10].

	Fixed-Dose Combination Therapy		Comparator			
Study	Events	Total	Events	Total	Weight	Relative Risk (95% CI)
UMPIRE 2013 [6]	829	1,002	621	1,002	31.0%	1.33 (1.26, 1.41)
IMPACT 2014 [9]	208	256	119	257	23.8%	1.75 (1.52, 2.03)
Kanyini GAP 2014 [8]	213	311	143	312	23.9%	1.49 (1.30, 1.72)
FOCUS 2014 [7]	169	350	133	345	21.3%	1.25 (1.05, 1,49)
**Overall**	**1,419**	**1,919**	**1,016**	**1,916**	**100%**	**1.44 (1.26, 1.65)**

Abbreviations: FOCUS, Fixed-Dose Combination Drug for Secondary Cardiovascular Prevention; IMPACT, IMProving Adherence using Combination Therapy; MI-FREE, Post-Myocardial Infarction Free Rx Event and Economic Evaluation; UMPIRE, Use of a Multidrug Pill In Reducing Cardiovascular Events

Adverse events were more common in participants who were randomized to fixed-dose combination therapy (30% versus 24%, RR = 1.20 [95% CI: 9% to 30%]), but some comparator groups included participants receiving placebo or usual care [5]. The three most common adverse events were elevated liver enzyme levels, cough, and myalgias. Using the Grading of Recommendations Assessment, Development and Evaluation (GRADE) framework, the quality of the overall evidence supporting fixed-dose combination therapy to improve adherence is high based on the consistency and precision of effect, low risk of bias (including reporting bias), and indirectness of evidence [10]. The level of recommendation would be strong based on the quality of evidence, the balance between desirable and undesirable effects, uncertainty between values and preferences, and cost.

## Current Availability and Regulatory Status

The current availability and regulatory approval of fixed-dose combinations that include aspirin, a statin, and at least one blood pressure lowering drug are detailed in Table 2. Approval of a polypill has yet to be granted by the Food and Drug Administration or European Medicines Agency, though the Food and Drug Administration’s Cardiovascular and Renal Drugs Advisory Committee met publicly in September 2014 to discuss the potential utility of fixed-dose combinations of aspirin, a statin, and blood-pressure-lowering drugs for secondary prevention of cardiovascular disease.

**Table 2:** Manufacturers and regulatory status of fixed-dose combinations for cardiovascular disease prevention [10].

Manufacturer	Combination	Current Regulatory Status
Ferrer Internacional, Spain	Trinomia (aspirin 100 mg + ramipril 2.5 mg, 5 mg, or 10 mg + simvastatin 40 mg or atorvastatin 20 mg)	Simvastatin version: Argentina, Dominican Republic, El Salvador, Guatemala, Honduras, Nicaragua, and México. Atorvastatin version: Austria, Belgium, Bulgaria, Czech Republic, Finland, France, Germany, Greece, Ireland, Italy, Poland, Portugal, Romania, Spain, and Sweden
Cadila Pharmaceuticals, India	Polycap (aspirin 100 mg + ramipril 5 mg + atenolol 50 mg + hydrochlorothiazide 12.5 mg + simvastatin 20 mg)	India and Zambia
Cipla, India	Aspirin 75 mg + losartan 50 mg + atenolol 50 mg + atorvastatin 10 mg. Aspirin 75 mg + losartan 25 mg + amlodipine 2.5 mg + atenolol 50 mg + simvastatin 40 mg for the polypill investigators (7)	India
Dr. Reddy’s Laboratory India (License held by the George Institute for Global Health)	Red Heart Pill 1 (aspirin 100 mg + lisinopril 10 mg + atenolol 50 mg + simvastatin 40 mg). Red Heart Pill 2 (aspirin 100 mg + lisinopril 10 mg + hydrochlorothiazide 12.5 mg + atenolol 50 mg + simvastatin 40 mg)	Not currently available

Long-term outcome trials would certainly influence adoption of fixed-dose combination for cardiovascular disease secondary prevention, but no such trials were performed before widespread adoption of combination therapy for HIV or inclusion of combination therapy for antimalarials on the WHO’s Model List of Essential Medicines. Even combination blood-pressure-lowering therapy has received regulatory approval based on pharmacokinetic and pharmacodynamics studies, yet the bar seems higher for polypills. In fact, two recent (2012 and 2014) applications to add fixed-dose combination therapy to the WHO’s Model List of Essential Medicines for secondary prevention of cardiovascular diseases have been unsuccessful because of the lack of outcome data [10,12]. However, the use of different thresholds for acceptance of combinations of drugs to the Model List is inconsistent and concerning, particularly for drugs that are widely approved and used to prevent and control the leading cause of death globally.

## Pragmatic Steps to Increase Polypill Availability and Uptake

Despite being approved and available in more than 20 countries, widespread penetration of the polypill has not been reported. Five near-term, pragmatic issues should be readily addressed by clinicians, researchers, public health experts, industry, patients, and guideline writers to help increase the availability and uptake of fixed-dose combination therapy.

### 1. Forsake the Age-Only Screening and Mass Treatment Approach Outlined by Wald and Law and Focus on Secondary Prevention

While the promise of the Polypill as proposed by Wald and Law [3] was captivating, the age-only screening and mass treatment approach does not have the scientific or sociopolitical capital to proceed. The effect of ongoing advocacy for this radical approach is uncertain, particularly given concerns about mass medicalization. Regardless, participants in all fixed-dose combination trials experience not only side effects but also serious adverse events, which were as high as one out of every three participants in the IMPACT trial, for example [9]. Many of these serious adverse events are from noncardiovascular causes, highlighting the limitations of framing fixed-dose combination as a mass treatment approach. Further, because rates of medication disutility, which represents the cost or inconvenience of taking a medication, vary widely [13], it seems unlikely that mass treatment would be accepted by many populations in the near term. On the other hand, individuals with prevalent cardiovascular disease clearly benefit from each of the individual components of fixed-dose combination therapy in the absence of contraindications to individual components of a polypill. Polypills have the potential to provide clinicians and patients, particularly high-risk individuals with low adherence, an additional tool to prevent and control cardiovascular diseases. Building an evidence base to demonstrate whether or not polypills can be successfully implemented for secondary prevention will be an important step in gaining the scientific capital to proceed.

### 2. Successful Pricing Models Need to Be Developed and Implemented

The transfer of licensure for Dr. Reddy’s Lab’s Red Heart Pills to the George Institute for Global Health was a major signal that pharmaceutical manufacturing companies have not widely developed successful pricing models, particularly on the heels of three large, well-conducted trials of the Red Heart Pill [6,8,9]. Other manufacturers like Ferrer, Cadila, or Cipla might use sliding scales based on country-level per capita income. Advanced bulk purchasing has also been described as one strategy to spur investment into fixed-dose combination therapy [14], but no such arrangements, whether by national governments, large insurance companies, or even organizations that emphasize logistics, such as Médecins Sans Frontières, have yet been made.

### 3. Patients and Payers Have Not Been Sufficiently Engaged for Mobilizing Community-Level Support, Particularly among Patients with Prevalent Cardiovascular Disease

Survey data from United States general physicians suggest that while many have reservations about the lack of flexibility of fixed-dose combination therapy, approximately 80% respond that they would use fixed-dose combination therapy for high-risk patients [15]. In concert with strategies to increase awareness and eventual prescription of fixed-dose combination among clinicians, strategies to engage and activate patients with prevalent cardiovascular disease would be useful. While activism within cardiovascular medicine is distinct from activism within other disease states (e.g., HIV), patients with prevalent cardiovascular disease would make natural and powerful allies. Further, payers such as governments and private insurers would seem to have much to gain from improved adherence and outcomes by using fixed-dose combination therapy.

### 4. Current Statin Dose Does Not Match Guidelines for Secondary Prevention of Cardiovascular Diseases in Most Nonelderly Patients

The 2013 American Heart Association/American College of Cardiology cholesterol guideline recommended a high-intensity statin for nonelderly patients with prevalent cardiovascular disease [16]. However, no currently available fixed-dose combination therapy includes a moderate dose of a high-intensity statin. While data from the SPACE collaboration demonstrate no difference in total and low-density lipoprotein (LDL) cholesterol in patients receiving a combination with a moderate statin compared with usual care, the perception that higher-intensity statins are not available in fixed-dose combinations will lead to lower uptake by clinicians for cardiovascular disease secondary prevention. Future fixed-dose combinations that include a moderate dose of a high-intensity statin will be more likely to be used by clinicians, even if the incremental benefit of statin titration is less than the proportional increase in dose.

### 5. Guideline Writers from Multiple Disciplines Should Include Fixed-Dose Combination Therapy as a Strategy to Improve Adherence

The majority of cardiovascular clinical practice guidelines are based on expert opinion [17], yet there is high-quality evidence supporting the use of fixed-dose combination therapy to improve adherence for cardiovascular disease prevention. As in blood pressure guidelines [18], fixed-dose combination therapy should be added to secondary prevention clinical practice guidelines as a strategy to increase adherence. Amidst other potential strategies, such as payment of drugs (MI-FREEE trial [19]) and nurse-based medication reconciliation [20], fixed-dose combination therapy has one of the largest relative effects with the lowest cost and greatest potential for scalability. However, clinicians may be reluctant to offer fixed-dose combination therapy outside of clinical norms, which are codified through clinical practice guidelines.

## Conclusions

Fixed-dose combination therapy appears to be following a typical hype-cycle curve of innovation wherein a peak of inflated expectations is followed by a trough of disillusionment, a slope of enlightenment, and a plateau of productivity [21]. While it is not a panacea, fixed-dose combination remains a promising pragmatic strategy to improve adherence and, eventually, outcomes that should continue to be researched so that its implementation will maximize benefit and minimize harm.

## Abbreviations

ACE: angiotensin-converting enzyme
FOCUS: Fixed-Dose Combination Drug for Secondary Cardiovascular Prevention; GRADE, Grading of Recommendations Assessment, Development and Evaluation; IMPACT, IMProving Adherence using Combination Therapy
LDL: low-density lipoprotein
MI-FREE: Post-Myocardial Infarction Free Rx Event and Economic Evaluation
RR: relative risk
SPACE: Single Pill to Avert Cardiovascular Events
UMPIRE: Use of a Multidrug Pill In Reducing Cardiovascular Events
